# Individual differences in approach-avoidance aptitude: some clues from research on Parkinson’s disease

**DOI:** 10.3389/fnsys.2015.00043

**Published:** 2015-03-24

**Authors:** Alberto Costa, Carlo Caltagirone

**Affiliations:** ^1^Department of Clinical and Behavioural Neurology, IRCCS Santa Lucia FoundationRome, Italy; ^2^Dipartimento di Medicina dei Sistemi, Rome University Tor VergataRome, Italy

**Keywords:** approach-avoidance, dopamine systems, Parkinson’s disease, personality, motivation disorders, cognitive functioning, executive abilities

## Abstract

Approach and avoidance are two basic behavioral aptitudes of humans whose correct balance is critical for successful adaptation to the environment. As the expression of approach and avoidance tendencies may differ significantly between healthy individuals, different psychobiological factors have been posited to account for such variability. In this regard, two main issues are still open that refers to (i) the role played by dopamine neurotransmission; and (ii) the possible influence of cognitive characteristics, particularly executive functioning. The aim of the present paper was to highlight the contribution of research on Parkinson’s disease (PD) to our understanding of the above issues. In particular, we here reviewed PD literature to clarify whether neurobiological and neuropsychological modifications due to PD are associated to changes in approach-avoidance related personality features. Available data indicate that PD patients may show and approach-avoidance imbalance as documented by lower novelty-seeking and higher harm-avoidance behaviors, possibly suggesting a relationship with neurobiological and neurocognitive PD-related changes. However, the literature that directly investigated this issue is still sparse and much more work is needed to better clarify it.

## Introduction

Actively seeking contact with rewarding stimuli and avoiding unpleasant conditions in the environment are critical in the functional adaptation of humans to their life context. Indeed, people learn early that in some conditions they have to maintain an approach attitude to pursue a desired goal and in other conditions they have to inhibit the tendency to move toward an object or a person to avoid negative outcomes. This implementation balance in approach-avoidance operations should allow the formation of behavioral modules at the level of the disposition to act, which represent the pre-conditions for obtaining correct knowledge of the world and one’s own limits, successful access to resources and, at the same time, provide for one’s own safety.

Within a psychobiological framework it has been posited that the activity of two main motivation systems modulates the approach-avoidance aptitude of an individual: the behavioral activation system and the behavioral inhibition system (Reinforcement Sensitivity Theory; Gray, 1970; Pickering and Gray, 2001). The first system was considered to mediate behavior related to gratifying conditions or potential positive outcomes of a situation and to be specifically sensitive to rewarding or non-punishing stimuli and to promote active searching for potentially rewarding conditions. By contrast, the behavioral inhibition system was considered particularly sensitive to punishment and non-rewarding stimuli. It modulated behavior by inhibiting appetitive responses and increasing arousal in order to improve attention to salient and relevant stimuli, e.g., potentially harmful stimuli, in the environment. The predominant activity of one of the two above systems was considered to lead to greater or even exclusive expression of behavioral moduli related to approach or, alternatively, to avoidance aptitudes, thus determining an individual’s stable dispositional response mode to external stimuli.

Recent findings deriving from both animal (mammals) models and human studies suggest that the activity of the above-mentioned motivational systems and, thus, the degree to which approach and avoidance behaviors can be expressed in an individual, depends on the variable effects of both biological and psychosocial factors. In particular, results of studies with mammals show that the approach-avoidance aptitude may be modulated by the central activity of the neuropeptides oxytocin (OT) and arginine vasopressin (AVP) in target brain regions (Young, 2002); interaction with the dopamine reward system was also suggested (Skuse and Gallagher, 2009). Human data document that in healthy subjects personality traits and some cognitive processes may be related to the likelihood of adopting approach or avoidance behavior (Rettew et al., 2006; Spielberg et al., 2011). Finally, in persons suffering from psychopathological disorders, the approach-avoidance related motivational systems may show differential sensitivity to environmental stimulation (Muris et al., 2001; Hirano et al., 2002; Mitchell and Nelson-Gray, 2006). In view of the above observations, interest has recently been centered on individual differences in approach-avoidance behavior and on the possible role played by the interaction between the different psychobiological factors in moderating its expression.

### Aims of the Review

The purpose of this paper was to highlight findings deriving from research in individuals with PD that might contribute to clarifying the factors related to individual differences in approach and avoidance aptitude. In particular, we here reviewed PD literature to clarify whether neurobiological and neuropsychological modifications due to PD are associated to changes in approach-avoidance related personality features. We focused on this issue for three main reasons: First, PD clinical manifestations are primarily a consequence of dopamine dysfunction in neural networks whose activity is considered important for sustaining the activity of behavioral motivation systems (Young, 2002; Calabresi et al., 2006; Laricchiuta et al., 2014). Second, some data suggest that PD patients develop personality characteristics and psychopathological disorders associated with avoidance behavior (Meyer et al., 1999; Muris et al., 2001). Third, PD patients frequently present neuropsychological disorders involving cognitive functions that are critical for sustaining goal-directed behavior (Halliday et al., 2014). These three points will be discussed by focusing mainly on the results of studies that suggested a potential relationship between the modifications occurring during the course of PD and the expression of various aspects of approach and avoidance behavior.

## Methods

The studies were searched using electronic database Medline and PsychoInfo in a period including the first months of 2014. In both databases the same following keywords were used: Approach-avoidance; Dopamine systems; Parkinson’s disease; Personality; Motivation Disorders; Cognitive functioning; Executive abilities. The studies included in the review should investigate, in PD patients, personality traits that could be related to approach-avoidance aptitude and their relationship with neurobiological and neuropsychological variables. A list of the studies that were considered with a description of main characteristics and results is reported in Table 1.

**Table 1:** **The table summarizes the results of main studies investigating approach-avoidance related functioning in individuals with Parkinson’s disease (PD)**.

Studies	Study design	Sample size (case/controls)	Personality measures	Results referring to the PD group
Menza et al. (1993)	Cross-sectional	51 PD; 31 controls with rheumatologic diseases	TPQ	Reduced Novelty seeking
Menza et al. (1995)	Cross-sectional	9 PD	TPQ	Significant correlation between 6-[18F]fluorodopa uptake in the caudate nucleus and novelty seeking
Jacobs et al. (2001)	Cross-sectional; Case-control	122 PD; 122 HCs	TPQ	Increased harm avoidance
Kaasinen et al. (2001)	Cross-sectional; Case-control	61 PD (47 underwent PET examination); 45 healthy subjects	TCI; KSP	Reduced novelty seeking; increased harm avoidance; significant association between increased harm avoidance and 6-[18F]fluorodopa uptake in the caudate nucleus
Tomer and Aharon-Peretz (2004)	Cross-sectional; Case-control	40 PD; 17 HCs	TPQ	Reduced novelty seeking; increased harm avoidance
Kaasinen et al. (2004)	Cross-sectional	28 PD	TCI	Negative correlation between novelty seeking score and insular cortex D_2_ receptors availability
McNamara et al. (2008)	Cross-sectional; Case-control	44 PD; 17 controls with chronic disease	TCI	Increased harm avoidance; Inverse correlation between verbal fluency and harm avoidance rates.
Bódi et al. (2009)	Longitudinal; Case-control	48 PD; 20 HCs	TCI	Reduced novelty seeking; novelty seeking improvement after dopamine treatment
Volpato et al. (2009)	Cross-sectional	25 PD	BFAC	Significant association between alternating verbal fluency and openness to experience factor
Arabia et al. (2010); Bower et al. (2010)	Longitudinal	6,822 persons without PD at baseline; 156 developed PD at follow-up	MMPI	Significant association between anxiety symptoms and the risk of PD
Koerts et al. (2013)	Cross-sectional; Case-control	43 PD; 25 HCs	TCI	Higher harm avoidance; Cognitive flexibility predicts reward dependence
Damholdt et al. (2014)	Cross-sectional	409 PD	Neo-FFI	Reduced extraversion rates associated with depression

*HCs: Healthy control subjects; PET: Positron emission tomography; TPQ: Tridimensional Personality Questionnaire (Cloninger, 1987); TCI: Temperament and Character Inventory (Cloninger et al., 1993); KSP: Karolinka Scales of Personality Questionnaire (Schalling et al., 1987); BFAC: Big Five Adjective Checklist (Caprara et al., 2002); MMPI Minnesota Multiphasic Personality Inventory (Dahlstrom et al., 1972); Neo-FFI: Neo-Five Factor Inventory (Costa and McCrae, 1992)*.

## Neurobiological Mechanisms of Approach-Avoidance Behavior and PD: Evidence of an Overlap

### Neurobiologic Correlates of Approach-Avoidance Behavior: Evidence from Non-PD Studies

Enter et al. (2012) found that dopamine transporter (DAT1) polymorphisms were related to different approach-avoidance behaviors when healthy adults were assessed using a task that had stimuli with emotional social valence (i.e., human faces). In particular, these authors demonstrated that, compared with DAT1 10-repeat homozygote carriers DAT1 9-repeat carriers showed an increased effect of the presented stimuli (happy and angry faces) in approach-avoidance responses. This finding suggests that the motivational behavioral systems of these subjects are more sensitive. The DAT is involved in dopamine reuptake in the striatum and the DAT1 9-repeat carriers have been reported to have lower levels of DAT than individuals with 10-repeat alleles, which indicates that these subjects have higher dopamine concentrations in the striatum (Heinz et al., 2000). Furthermore, in a recent functional magnetic resonance imaging (fRMI) study in healthy young subjects, Simon et al. (2010) documented greater ventral striatal and mesial orbito-frontal cortex activation when individuals who showed a high expression of reward-seeking behavior actually received rewards. By contrast, they found less ventral striatal activation when subjects who were more prone to inhibit appetitive behavior received a reward (Simon et al., 2010). These findings provide evidence in line with previous data from animals models that dopamine neurotransmission in neural networks (including the striatal structures) is critically involved in the modulation of motivation behavior (For a review on animal models see, Hoebel et al., 2007). In particular, based on findings suggesting that dopamine activity would promote appetitive behavior (e.g., moving toward external stimuli, reward seeking), whereas acetylcholine would mainly enhance behavioral inhibition and aversive responses (Mark et al., 1995; Avena et al., 2006), Hoebel et al. (2007) proposed that dopamine interacts with acetylcholine in the ventral striatum (i.e., in the nucleus accumbens) to maintain a functional balance between approach and avoidance tendencies.

### Neurobiological Modifications in PD

PD is a well-known neurological disease that is primarily characterized by dysregulation of the nigro-striatal, mesolimbic and the mesocortical dopaminergic brain systems. (Owen, 2004; Dickson et al., 2009). More specifically, degeneration of the dopamine cells in the midbrain leads to precocious and severe dopamine depletion in the striatum, which first involves the rostrodorsal extent of the head of the caudate nucleus and, later, the ventral tegmental neurons that project to more ventral parts of this structure and to prefrontal and limbic regions (Yeterian and Pandya, 1991; Agid et al., 1993; Costa et al., 2009). In fact, in addition to movement disorders PD patients often display cognitive-behavioral deficits (Robbins and Cools, 2014). Although the role of dopamine brain transmission in causing cognitive-behavioral disorders in PD has not yet been completely clarified, in the early phase of the disease cognitive deficits are considered due to an imbalance between phasic dopamine activity in the dorsal striatum and tonic dopamine activity in the prefrontal cortex, which leads to reduced efficiency of flexibility processes (i.e., updating and set-shifting) (Cools, 2006; Cools and D’Esposito, 2011). With disease progression and the parallel greater involvement of dopamine transmission in the ventral striatum and the dopamine projections to the other structures of the mesolimbic system, reduced ability to decode and use environmental stimulation (e.g., reinforcers) to adopt functional behavior, and altered emotional processing and declarative memory disorders are observed. The hypothesis was also advanced that the disrupted equilibrium between the activity of dopamine and acetylcholine, which occurs in the striatum, could account for some of the cognitive-behavioral manifestations of PD (Calabresi et al., 2006). As stated above, dopamine projections to striatal structures are primarily affected in PD, thus causing a decrease of dopamine activity and, likely, a parallel increase of cholinergic tone. According to Calabresi et al. (2006) the altered dopamine-acetylcholine equilibrium could affect synaptic mechanisms of long-term potentiation and depression and of synaptic depotentiation, in some way modifying frontalstriatal interconnections and causing learning and executive disorders.

The above mentioned evidence suggests that PD may precociously cause functional and structural changes in frontal-striatal regions whose activity is supposed to be responsible for the modulation of approach and avoidance responses in animals as well as in humans. Thus, PD is an interesting natural human model for investigating the psychobiological mechanisms involved in learning and sustaining these behavioral aptitudes.

## Do the Personality and Psychopathological Features of PD Indicate an Approach-Avoidance Imbalance?

In the previous section we suggested that the neuropathological processes of PD might affect the functioning of brain circuitries involved in the mediation of approach and avoidance tendencies. This leads to the key question of whether these two main aspects of the behavioral motivational systems are impaired in PD patients. Some clinical reports are in line with this idea. In fact, a large proportion of PD patients suffer from depressive disorders and apathy (Aarsland et al., 2011; Martínez-Horta et al., 2013), which have been shown to be associated with a significant decrease of appetitive and self-initiated behaviors also in PD (Costa et al., 2006; Martínez-Horta et al., 2013; Damholdt et al., 2014; Spielberg et al., 2014). Anxiety symptoms, which are associated with avoidance behavior, particularly in the context of social interactions (Wong and Moulds, 2011), are also frequently described in these patients (Sagna et al., 2014). An opposite behavioral pattern, characterized by excessive attraction to rewarding stimuli, is observed in some PD individuals who develop impulse control disorders especially in response to the administration of dopamine therapy (Callesen et al., 2013).

More direct evidence of an imbalance between the behavioral activation and inhibition systems comes from research on the personality functioning of PD patients. These data document that personality traits such as novelty seeking, which mainly refers to the propensity towards active exploration in response to novel stimuli and the avoidance of frustration (Cloninger, 1987), are expressed to a lesser extent in PD patients compared to controls without neurologic diseases (Menza et al., 1993; Bódi et al., 2009; for a review see Poletti and Bonuccelli, 2012). By contrast, the personality trait of harm avoidance, which is characterized by the inclination to adopt a passive avoidance behavior, was found to be much more present in PD patients than in controls (Jacobs et al., 2001; Tomer and Aharon-Peretz, 2004; McNamara et al., 2008; Koerts et al., 2013). Other studies reported evidence of reduced extraversion in PD patients (Damholdt et al., 2014), which probably indicates their low aptitude for approach social interactions (McCrae and Costa, 1997).

### Personality Modifications May Predate Clinical Manifestation of PD

It was also hypothesized that personality changes might occur in a pre-clinical phase of PD. This hypothesis was mainly grounded on the observation that the presence of a personality with low novelty-seeking functioning, rigidity and caution predates the onset of extrapyramidal symptoms (for a review see Menza, 2000). Nevertheless, few longitudinal studies have been conducted to investigate this hypothesis. In this regard, some interesting data were reported by Bower et al. (2010) and by Arabia et al. (2010) in a cohort study in which more than 6,800 persons were followed for four decades. The Minnesota Multiphasic Personality Inventory, a validated psychometric test that investigates psychological disorders (Dahlstrom et al., 1972), was used to assess personality. The authors did not find a clear relationship between the constructs of introversion and extroversion and the risk of PD. However, results showed that an anxious personality, as assessed by the psychoastenia scale, is associated with a higher risk of developing PD with a hazard ratio of 1.63. This finding is quite interesting for our discussion because there are reports that anxiety symptoms are associated with avoidance behavior in both human studies (Muris et al., 2001; Wong and Moulds, 2011) and animal models (Toth and Neumann, 2013). Evidence that the neurobiochemical alterations of PD occur years before clinical manifestation of the disease also support the idea of a correlation with these personality changes. Nevertheless, findings from studies that directly correlated the personality changes and neurobiological modifications of PD are still sparse.

### Relationship between Personality Changes and Neurobiological Modifications in PD

#### Findings Indicating a Positive Association

Bódi et al. (2009) documented a significant relationship between dopamine stimulation and novelty seeking in drug-naïve PD patients. In particular, they investigated the effect of dopamine agonist administration on different personality traits (i.e, novelty seeking, harm avoidance, reward dependence and persistence) and on reward-learning using a feedback-based task. Patients underwent two assessments in which the examiners were blind to personality measures, test results and medication conditions, the first without taking any medication and the second after a 12-week period of treatment with the D_2_ and D_3_ dopamine receptor agonists pramipexole and ropinirole. At the first assessment PD patients showed significantly lower novelty-seeking and reward-learning scores than healthy controls. The second assessment showed that dopamine intake significantly improved PD patients’ novelty-seeking scores and reward-learning performance so that they could no longer be distinguished from healthy controls on these measures (Bódi et al., 2009).

Tomer and Aharon-Peretz (2004) reported more complex results. They showed that left-right side asymmetry of dopamine-related pathology may differentially affect personality functioning in PD patients. In fact, findings from this study suggest that in these patients dopamine loss in the left hemisphere is associated with reduced novelty- seeking behavior while higher harm avoidance is related to dopamine loss in the right hemisphere (Tomer and Aharon-Peretz, 2004). These findings are in line with those of a previous PET study in PD patients in which Menza et al. (1995) demonstrated that 6-[18F]fluorodopa uptake in the caudate nucleus (of the left hemisphere) correlated with novelty-seeking scores.

Above observations are congruent with previous evidence in people without neurological disorders of the relevant role of dopamine transmission in the striatum in modulating sensitivity to reward- and novelty-seeking behavior (Leyton et al., 2002), and would sustain the general hypothesys that novelty seeking is strongly related to dopamine transmission (Cloninger, 2000; but see Paris, 2005 for a critical review). Also interestingly, the above findings are congruent with the hypothesis of brain hemispheric asymmetry in mediating activation and inhibition behavioral systems, with the left hemisphere more associated with approach and the right hemisphere with avoidance (Spielberg et al., 2013).

#### Studies Documenting a Non-Linear Association between Personality Features and PD Related Neurobiological Changes

Partially divergent results in respect to those above discussed were reported in other studies with PD patients. Indeed, Kaasinen et al. (2004) showed an inverse correlation between novelty-seeking scores and dopamine receptor availability in the insula, a brain region highly interconnected with the striatum and suggested to be involved in different cognitive and emotional processes in PD (Christopher et al., 2014). The finding by Kaasinen et al. (2004) indicates that the likelihood of PD patients expressing a higher novelty-seeking aptitude corresponds with lower dopamine activity in this structure. In another independent PET study Kaasinen et al. (2001) also demonstrated that in unmedicated PD patients novelty-seeking scores did not correlate with 6-[18F]fluorodopa uptake in any of the target brain regions. Instead, higher harm avoidance scores were positively correlated with 6-[18F]fluorodopa uptake in the caudate nucleus.

*In summary*, an imbalance between the activity of behavioral activation and inhibition systems, characterized by lower approach and higher avoidance tendencies, seems to be present in PD patients (see Table 1 for a synthesis of the results of the main studies). This is supported by observations of their reduced novelty seeking, sensitivity to reward and self-initiated behavior. However, results on the potential role played by the neurobiological changes of PD and this hypothesized imbalance are inconclusive. One limit of most studies investigating this issue in PD patients is the use of self-rating psychometric tools that require self-judgment of one’s own characteristics. Further studies using more objective, performance-based paradigms, which specifically assess approach and avoidance behaviors, would be more informative. For instance, to better understand the role of dopamine neurotransmission in target brain regions on these processes, a functional magnetic resonance protocol could be used to investigate how dopamine administration/withdrawal modulates neural activity in PD patients while they perform approach-avoidance procedures. Future investigations should also take into account several potentially confounding individual clinical (e.g., disease duration, disease severity, side of onset, pattern of movement disorders) and cognitive (e.g., presence of dementia, mild cognitive impairment or attention disorders) characteristics of the disease.

## Are the Executive Disorders in PD Potentially Related to Approach-Avoidance Tendencies?

### Results Evidencing an Association between Approach-Avoidance Behavior and Executive System in Individuals without PD

The interaction between approach and avoidance motivational systems and executive functions appears to be critical for the maintenance of goal pursuit and, thus, for the implementation of adaptive behavior (for a review see Spielberg et al., 2008, 2013). This interaction has been observed in both behavioral and neuroimaging investigations. Gray (2001) showed that the induction of approach and withdrawal motivation differentially affected subjects’ performance on verbal and spatial working memory tasks. In a subsequent study Spielberg et al. (2011) showed that trait motivation modulated dorsolateral prefrontal cortex activity while healthy subjects were performing a selective attention task (i.e., the Stroop color-word task). Specifically, in a fMRI protocol, these authors explored changes in neural activity as a function of subjects’ scores on questionnaires investigating approach and avoidance temperament. Results showed a significant positive correlation between approach temperament scores and activation in the left superior and middle frontal gyri. A positive association was also found between avoidance scores and activation in the middle frontal gyrus (bilaterally) and the left superior frontal gyrus (Spielberg et al., 2011).

Similar results indicating an interaction at the level of the prefrontal cortex between motivation and executive abilities were previously reported by Pochon et al. (2002) and by Taylor et al. (2004). Indeed, the prefrontal cortex has been consistently found to be highly involved in the mediation of several executive abilities (e.g., planning, working, multitasking, prospective memory) and it is considered fundamental for the correct organization of information and goal-directed behavior (Miller and Cohen, 2001; Burgess et al., 2011; Yuan and Raz, 2014). A recently proposed integrated view accounts for the interaction between approach-avoidance traits, state motivation and executive skills in coherently pursuing internal goals (Spielberg et al., 2013).

On the basis of the above observations it can be hypothesized that the qualitative characteristics of executive functioning influence the expression of individual approach and avoidance tendencies. This hypothesis is corroborated by the clinical manifestation of brain damage involving the prefrontal cortices. These patients often present cognitive and behavioral signs—such as a decrease in goal-directed behavior, apathy and disinhibition—that could be related to an imbalance between activation and inhibition systems.

### Results from Studies with PD Patients

We previously mentioned that PD patients present cognitive disorders early in the disease course, which are primarily related to dopamine dysfunction in the frontal-striatal circuitries (Costa et al., 2014a), with convergent evidence documenting their reduced ability to perform tasks sensitive to executive functions (Dirnberger and Jahanshahi, 2013; Kudlicka et al., 2013; Robbins and Cools, 2014). In particular, set-shifting and updating efficiency appear to be reduced early, likely affecting their ability to successfully maintain a goal- directed behavior (Cools, 2006; Cools and D’Esposito, 2011). In fact, these patients have difficulty in performing planning and multitasking tests and in spontaneously retrieving the intention to perform planned actions (Owen, 2004; Kliegel et al., 2011; Costa et al., 2014b,c).

Based on above observations documenting both an association between approach and avoidance motivational systems and executive functions in non-PD individuals and, in PD patients, a decreased efficiency of the executive system, we could hypothesize, in the latters, the existence of a relationship between executive dysfunctioning and approach-avoidance imbalance. Some findings are in line with these hypothesis (in Table 1 a synthesis of main results is reported).

McNamara et al. (2008) reported, in 44 PD patients without dementia, a significant inverse correlation between harm avoidance rates, as assessed by means of the Temperament and Character Inventory, and a measure of cognitive flexibility and strategic access to information (i.e, verbal fluency). In an other study, Volpato et al. (2009) administered to 25 PD patients without dementia the Big Five Adjective Checklist to examine different personality traits, and the Tower of London test and Alternating Fluency test to investigate planning and set-shifting, respectively. The authors found a significant correlation between the personality factor of openness to experience and PD patients’score on alternating fluencies, thus indicating a significant relationship between this personality factor and cognitive flexibility processes.

In a more recent study with 43 PD patients without dementia, Koerts et al. (2013) investigated the relationship between different components of executive functions (cognitive flexibility, inhibition, working memory, planning and verbal fluency) and various aspects of personality functioning (novelty seeking, harm avoidance, persistence and reward dependence). Neuropsychological tests and questionnaires (the Temperament and Character Inventory) were administered to assess cognitive and personality features, respectively. The authors found that PD patients’ scores on cognitive flexibility measures significantly predicted reward dependence rates (Koerts et al., 2013).

*In summary*, taken together the above findings do not allow us to make firm conclusions about the nature of the relationship between executive functioning and approach-avoidance imbalance in PD. Indeed, the reported associations between some personality factors (i.e, reward dependance and openness to experience) and cognitive flexibility is undoubtedly interesting as neural activity in the frontal-striatal circuitries has been demonstrated to be critical for both reward-related behavior and cognitive flexibility (O’Doherty, 2004; Bódi et al., 2009; Macdonald and Monchi, 2011). However, a better method for investigating the relationship between executive functioning and approach-avoidance aptitude in PD patients would be to test the effect of cognitive training aimed at potentiating executive abilities on approach-avoidance related behavior. The observation of executive improvement combined with a change in approach-avoidance behavior would be clear evidence of a causal relationship. In this regard it should be noted that some cognitive training has been proposed which significantly enhances some components of executive functioning, e.g., cognitive flexibility, which, as we discussed here, may be associated with approach-avoidance tendencies also in persons with PD (Calleo et al., 2012; Costa et al., 2014b).

## Conclusions

Currently, the main issues in the study of individual differences in the expression of approach and avoidance behaviors are (i) the role played by dopamine neurotransmission; and (ii) the possible influence of cognitive characteristics, particularly executive functioning. Regarding the first point, there is some evidence that dopamine and its interaction with other neuromodulators at the level of the mesocorticolimbic networks affects these processes by modulating the learning of individual response modalities (Skuse and Gallagher, 2009). However, this evidence is mainly derived from results of studies that used animal models; in fact, human data are inconclusive. Regarding the second point, also in this case the results of some studies suggest the existence of a potentially bidirectional relationship between cognitive (executive) functioning and approach-avoidance behavior. Extant findings are, however, still sparse and not univocal.

Here we highlight the potentially relevant contribution of research on PD in clarifying above issues. In fact, PD is characterized early by dopamine loss in brain circuitries including the frontal-striatal, mesolimbic and mesocortical pathways, that are implicated in sustaining the functioning of motivational behavioral systems and executive processes.

Some evidence seem to support the idea that PD may be associated to an alteration of approach-avoidance behaviors, wherein it documents that PD patients show reduced novelty seeking and higher harm avoidance expression. However, the literature that directly investigated the relationship between PD patients’ neurobiological and neuropsychological modifications and approach-avoidance related personality functioning gave inconclusive results. Further research that overcomes the limits of previous studies (e.g., low sample size, clinical heterogeneity, heterogeneity of dopamine treatments, use of self-report questionnaires) are needed to further explore these important topics in PD.

## Conflict of Interest Statement

The authors declare that the research was conducted in the absence of any commercial or financial relationships that could be construed as a potential conflict of interest.
